# Allergic rhinitis and asthma among children and adolescents in Germany. Results of the cross-sectional KiGGS Wave 2 study and trends

**DOI:** 10.17886/RKI-GBE-2018-027

**Published:** 2018-03-15

**Authors:** Christina Poethko-Müller, Michael Thamm, Roma Thamm

**Affiliations:** Robert Koch Institute, Berlin, Department of Epidemiology and Health Monitoring

**Keywords:** ALLERGIC RHINITIS, ASTHMA, TRENDS, CHILDREN AND ADOLESCENTS, HEALTH MONITORING

## Background

Allergic diseases such as allergic rhinitis and bronchial asthma belong to the most common conditions suffered by children and adolescents. In many cases, the symptoms have a severe effect on the everyday lives of patients. Allergic rhinitis is an allergic inflammation of the nasal mucosa, and is accompanied by itchiness, sneezing attacks, increased secretion of mucus, and impaired nasal breathing. Frequently the condition also affects the eyes. Allergens ranging from pollen, fungi, epithelial animal tissue to house dust mites may all trigger symptoms. Bronchial asthma, in turn, is caused by a hypersensitivity of the bronchi to various compounds. This hypersensitivity causes reversible, sudden constriction of the bronchial system that leads to coughing, shortness of breath and wheezing. Asthma in the majority of children has an allergic cause [1, 2].

From the mid-20th century, the prevalence of allergic diseases in western industrialised nations saw a significant increase. The results of the international ISAAC study (International Study of Asthma and Allergies in Childhood) and the repeated examination of children at school-entry age in East and West Germany during the 1990s, revealed a further, albeit not so pronounced increase in Germany [3–5]. As far as the development over the past ten years can be assessed, the results from the baseline study of the German Health Interview and Examination Survey for Children and Adolescents (KiGGS) between 2003 and 2006 served as a benchmark for a comparison with the prevalence measured in KiGGS Wave 2 between 2014 and 2017.

## Indicators and methodology

The German Health Interview and Examination Survey for Children and Adolescents is part of the health monitoring programme undertaken at the Robert Koch Institute. It involves repeated cross-sectional surveys of children and adolescents aged between 0 and 17 that are representative of the German population (KiGGS cross-sectional study). After carrying out the baseline study as an interview and examination survey (2003-2006) and KiGGS Wave 1 as an interview-based survey (2009-2012), KiGGS Wave 2 took place between 2014 and 2017 as a combined examination and interview survey.

A detailed description of the methodology used in KiGGS Wave 2 can be found in New data for action. Data collection for KiGGS Wave 2 is completed in issue S3/2017 as well as KiGGS Wave 2 cross-sectional study – participant acquisition, response rates and representativeness in issue 1/2018 of the Journal of Health Monitoring [6, 7].

On the basis of data from the KiGGS baseline study and KiGGS Wave 2, this article reports the trends in the 12-month prevalence of allergic rhinitis and bronchial asthma in the group of children and adolescents aged between 3 and 17. In both survey waves parents were asked, whether a physician had ever diagnosed their children with the condition, whether the condition had occurred during the last twelve months and whether their child had taken any corresponding medication during the last twelve months. For the group of children and adolescents diagnosed with allergic rhinitis or atopic dermatitis with a positive allergy test result, the survey also compared parents’ answers regarding specific immunotherapy between the baseline study and KiGGS Wave 2.

**KiGGS Wave 2**

Second follow-up to the German Health Interview and Examination Survey for Children and Adolescents

**Data owner:** Robert Koch Institute

**Aim:** Providing reliable information on health status, health-related behaviour, living conditions, protective and risk factors, and health care among children, adolescents and young adults living in Germany, with the possibility of trend and longitudinal analyses

**Study design**: Combined cross-sectional and cohort study

**Cross-sectional study in KiGGS Wave 2**

**Age range:** 0-17 years

**Population:** Children and adolescents with permanent residence in Germany

**Sampling:** Samples from official residency registries - randomly selected children and adolescents from the 167 cities and municipalities covered by the KiGGS baseline study

**Sample size:** 15,023 participants

**KiGGS cohort study in KiGGS Wave 2**

**Age range:** 10-31 years

**Sampling:** Re-invitation of everyone who took part in the KiGGS baseline study and who was willing to participate in a follow-up

**Sample size:** 10,853 participants

**KiGGS survey waves**

► KiGGS baseline study (2003-2006), examination and interview survey
► KiGGS Wave 1 (2009-2012), interview survey
► KiGGS Wave 2 (2014-2017), examination and interview survey

More information is available at www.kiggs-studie.de/english

All calculations were carried out using a weighting factor that corrects for deviations within the sample from the population structure with regard to age in years, gender, federal state, nationality and parent level of education (Microcensus 2013 [8]).

This article reports prevalences with 95% confidence intervals (95% CI). A statistically significant difference between groups is assumed to have been demonstrated with p-values of less than 0.05 (once weighting had been applied and the survey design had been taken into account).

## Results

### Allergic rhinitis

In KiGGS Wave 2, the 12-month prevalence of physician-diagnosed allergic rhinitis in the 3 to 17 age group was 9.9% (95% CI 9.2-10.7) and has therefore remained nearly unchanged compared to the KiGGS baseline study (9.6%; 95% CI 9.0-10.1). Equally unchanged are the observed charateristic differences by gender and age, with prevalence higher for boys than for girls (KiGGS Wave 2: 11.9% vs. 7.9%) and a clear increase of prevalence with age for both genders.

### Asthma

In KiGGS Wave 2, the 12-month prevalence of physician-diagnosed bronchial asthma in the 3 to 17 age group was 4.0% (95% CI 3.5-4.5). Overall prevalence also has not changed significantly in comparison to the KiGGS baseline study (3.7%; 95% CI 3.3-4.1). Stratified by gender, prevalence among girls between the two survey points remained unchanged (3.0% vs. 3.1%) and increased slightly for boys (5.0% vs. 4.2%). This increase in prevalence is owed mainly to increases in the groups of boys aged 7 to 10 (5.7% vs. 4.1%) and 11 to 13 (7.1% vs. 5.7%).

### Specific immunotherapy

The proportion of children and adolescents who have been medically diagnosed with allergic rhinitis or atopic dermatitis including a positive allergy test result and subsequent specific immunotherapy has increased significantly in the 11 to 17 age group. During the KiGGS baseline study 24.3% (95% CI 21.3-27.6) of elder children and adolescents reported having undergone a specific immunotherapy treatment, in KiGGS Wave 2 this proportion had increased to 30.1% (95% CI 26.5-33.9).

## Discussion

For both allergic rhinitis and bronchial asthma, KiGGS Wave 2 results, when compared to the KiGGS baseline study, show that following the secular trends with a significant increase in the number of cases registered during the second half of the 20th century, there are strong indications of a stabilisation of this trend, albeit at a high level. Stratified by gender, the results indicate that the trends for girls and boys might be developing differently, particularly regarding bronchial asthma. Whereas the survey observed no changes in 12-month prevalence among girls between KiGGS baseline and KiGGS Wave 2, there has been a slight increase of prevalence for boys aged 7 to 13. The new results, however, give little reason for contentedness: allergic rhinitis still affects over one million children and adolescents aged between 3 and 17, and nearly half a million suffer from asthma.

The increase in the number of cases receiving specific immunotherapy treatment as the single causal therapy for elder children with allergic rhinitis or atopic dermatitis is positive. The guidelines developed jointly by the German Society for Allergology and Clinical Immunology (DGAKI) and other allergy associations on specific immunotherapy to treat allergic diseases recommend an early onset of therapy in particular at child and adolescent age [9] to reduce the risks for new sensitisation and asthma. Overall, early diagnosis and adequate care provision for allergic diseases are important not only for patients but also from a macro-economic perspective.

## References

[1] Pawankar R Sanchez-Borges M Bonini S (2013) Allergic rhinitis, allergic con-junctivitis, and rhinosinusitis. In: Pawankar R Canonica G Holgate S (Eds) World Allergy Organization (WAO) White Book on Allergy: Update 2013, WAO, Milwaukee, P. 27-33

[2] Wahn U Seger R Wahn V (2005) Pädiatrische Allergologie und Immunologie. Elsevier Urban & Fischer, München

[3] Bjorksten B Clayton T Ellwood P (2008) Worldwide time trends for symptoms of rhinitis and conjunctivitis: Phase III of the International Study of Asthma and Allergies in Childhood. Pediatr Allergy Immunol 19(2):110-124 17651373 10.1111/j.1399-3038.2007.00601.x

[4] Pearce N Ait-Khaled N Beasley R (2007) Worldwide trends in the prevalence of asthma symptoms: phase III of the International Study of Asthma and Allergies in Childhood (ISAAC). Thorax 62(9):758-766 17504817 10.1136/thx.2006.070169 PMC2117323

[5] Kramer U Link E Oppermann H (2002) Die Schulanfängerstudie in West- und Ostdeutschland (SAWO): Trends von Allergien und Sensibilisierungen 1991–2000. Gesundheitswesen 64(12):657-663 12516017 10.1055/s-2002-36461

[6] Hoffmann R Lange M Butschalowsky H (2018) KiGGS Wave 2 cross-sectional study – participant acquisition, response rates and representativeness. Journal of Health Monitoring 3(1):78-91. www.rki.de/journalhealthmonitoring-en (As at 15.03.2018) 10.17886/RKI-GBE-2018-032 PMC8848911 35586176

[7] Mauz E Gößwald A Kamtsiuris P (2017) New data for action. Data collection for KiGGS Wave 2 has been completed. Journal of Health Monitoring 2(S3):2-27. http://edoc.rki.de/oa/articles/revpaHQ3DqMU/PDF/25Pxmf2f-cHqRM.pdf (As at 27.09.2017) 10.17886/RKI-GBE-2017-105 PMC10291840 37377941

[8] Research Data Centres of the Federal Statistical Office and Statistical Offices of the Länder (2017) Microcensus,. 2013, own calculations. http://www.forschungsdatenzentrum.de/en/database/microcen-sus/index.asp (As at 20.11.2017)

[9] DGAKI-Arbeitsgruppe (2014) Leitlinie zur (allergen-)spezifischen Immuntherapie bei IgE-vermittelten Erkrankungen, (AWMF 061-004). Allergo J Int 23:282-31926120539

